# Author Correction: A HIF independent oxygen-sensitive pathway for controlling cholesterol synthesis

**DOI:** 10.1038/s41467-023-43699-w

**Published:** 2023-11-27

**Authors:** Anna S. Dickson, Tekle Pauzaite, Esther Arnaiz, Brian M. Ortmann, James A. West, Norbert Volkmar, Anthony W. Martinelli, Zhaoqi Li, Niek Wit, Dennis Vitkup, Arthur Kaser, Paul J. Lehner, James A. Nathan

**Affiliations:** 1 Cambridge Institute of Therapeutic Immunology & Infectious Disease (CITIID), Jeffrey Cheah Biomedical Centre, Department of Medicine, University of Cambridge, Cambridge, CB2 0AW UK; 2 Institute for Molecular Systems Biology (IMSB), ETH Zürich, Zürich, Switzerland; 3 DISCO Pharmaceuticals Swiss GmbH, ETH Zürich, Zürich, Switzerland; 4 Department of Biology, Massachusetts Institute of Technology, Cambridge, MA USA; 5 Department of Systems Biology, Columbia University, New York, NY USA; 6 Department of Biomedical Informatics, Columbia University, New York, NY USA; 7 Present Address: Ochre-Bio Ltd, Hayakawa Building, Oxford Science Park, Edmund Halley Road, Oxford, OX4 4GB UK; 8 Present Address: Biosciences Institute, Newcastle University, Herschel Building, Level 6, Brewery Lane, Newcastle upon Tyne, NE1 7RU UK; 9 Present Address: Tango Therapeutics, 201 Brookline Ave Suite 901, Boston, MA USA

**Keywords:** Ubiquitylation, Sterols, Cancer metabolism, Nutrient signalling

Correction to: *Nature Communications* 10.1038/s41467-023-40541-1, published online 09 August 2023

In this article the affiliation details for Anna S. Dickson were incorrectly given as ‘Present address: Discovery Sciences, BioPharmaceuticals R&D, AstraZeneca, Cambridge, UK’.

The original article has been corrected.

