# Author Correction: A HIF independent oxygen-sensitive pathway for controlling cholesterol synthesis

**DOI:** 10.1038/s41467-024-47041-w

**Published:** 2024-03-26

**Authors:** Anna S. Dickson, Tekle Pauzaite, Esther Arnaiz, Brian M. Ortmann, James A. West, Norbert Volkmar, Anthony W. Martinelli, Zhaoqi Li, Niek Wit, Dennis Vitkup, Arthur Kaser, Paul J. Lehner, James A. Nathan

**Affiliations:** 1 Cambridge Institute of Therapeutic Immunology & Infectious Disease (CITIID), Jeffrey Cheah Biomedical Centre, Department of Medicine, University of Cambridge, Cambridge, CB2 0AW UK; 2 Institute for Molecular Systems Biology (IMSB), ETH Zürich, Zürich, Switzerland; 3 DISCO Pharmaceuticals Swiss GmbH, ETH Zürich, Zürich, Switzerland; 4 Department of Biology, Massachusetts Institute of Technology, Cambridge, MA USA; 5 Department of Systems Biology, Columbia University, New York, NY USA; 6 Department of Biomedical Informatics, Columbia University, New York, NY USA; 7 Present Address: Ochre-Bio Ltd, Hayakawa Building, Oxford Science Park, Edmund Halley Road, Oxford, OX4 4GB UK; 8 Present Address: Biosciences Institute, Newcastle University, Herschel Building, Level 6, Brewery Lane, Newcastle upon Tyne, NE1 7RU UK; 9 Present Address: Tango Therapeutics, 201 Brookline Ave Suite 901, Boston, MA USA

**Keywords:** Ubiquitylation, Sterols, Cancer metabolism, Nutrient signalling

Correction to: *Nature Communications* 10.1038/s41467-023-40541-1, published online 09 August 2023

The original version of this Article contained errors in Figs. 2, 3, and 5. In the original Fig. 2e, the flow cytometry panel on the right (labelled “StD (24 hr) followed by 1% O_2_ (~16 hr)”), was inadvertently duplicated from the panel on the left (labelled “Concurrent StD and 1% O_2_ (~24 hr)”). In the original Fig. 3a, the flow cytometry panel on the right (labelled “Roxadustat”), was inadvertently duplicated from the panel on the left (labelled “DMOG”). In the original Fig. 5c, the labels did not properly communicate that both panels come from the same experiment and have the same controls. The following sentence has been added to the end of the legend for Fig. 5c: “The data depicted in the left and right panels originated from the same experiment and as such the control plots are the same in both.” Figures 2, 3, and 5 have been corrected in both the PDF and HTML versions of the Article.

The original version of the Supplementary Information associated with this Article contained an error in Supplementary Fig. 5. In the original Supplementary Fig. 5a, the labels did not properly communicate that all three panels come from the same experiment and have the same control. The following sentence has been added to the end of the legend for Supplementary Fig. 5a: “The data depicted in the three panels originated from the same experiment and as such the control plot is the same in all panels”. The HTML has been updated to include a corrected version of the Supplementary Information.

### Supplementary information

Supplementary Information

