# Supplementary material for: A HIF independent oxygen-sensitive pathway for controlling cholesterol synthesis
Source: Nat Commun. 2023 Aug 9;14:4816. doi: 10.1038/s41467-023-40541-1 (PMC10412576; doi:10.1038/s41467-023-40541-1)
Supplement: Supplementary file 1 — Supplementary Information File [file 41467_2023_40541_MOESM1_ESM.pdf]

Supplementary Information:

A HIF independent oxygen-sensitive pathway for controlling cholesterol synthesis

Supplementary Figures 1-7

Supplementary Tables 1-6

Supplementary References

Supplementary Fig.1: Hypoxia promotes SREBP2 degradation.

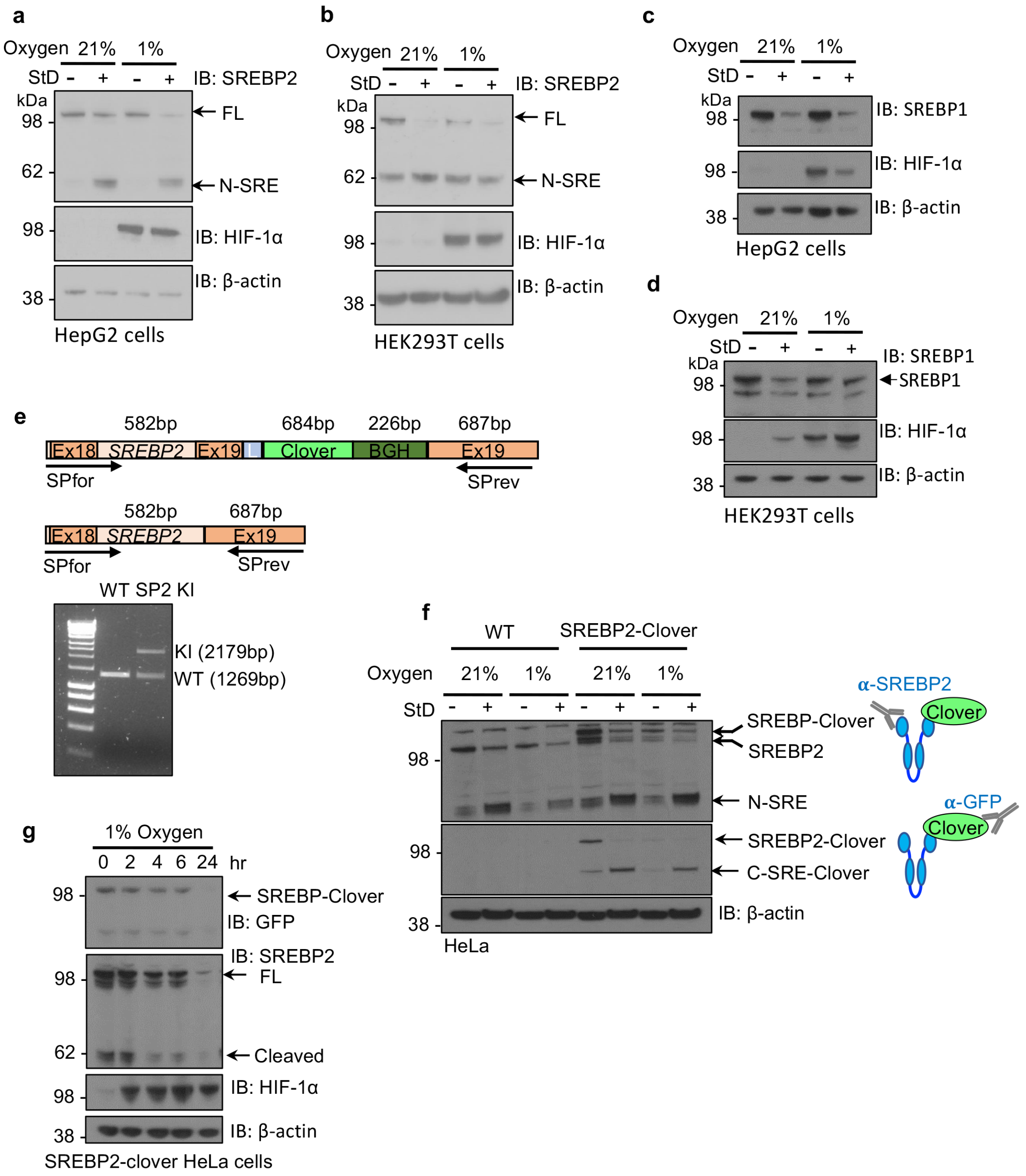

(**a, b**) SREBP2 processing in HepG2 cells (**a**) or HEK23T cells (**b**) treated with or without StD in 21% and 1% oxygen for 40 hr. Immunoblots representative of 3 independent experiments. (**c, d**) SREBP1 levels in HepG2 cells (**c**) or HEK23T cells (**d**) treated with or without StD in 21% and 1% oxygen for 40 hr. Immunoblots representative of 2 independent experiments. (**e**) PCR confirmation of Clover knock-in at the C-terminus of SREBP2. Genomic DNA was extracted from parental HeLa cells and clonal SREBP2-Clover knock-in cells, and amplified using the primers indicated. The presence of the 2179bp fragment confirmed incorporation of Clover to the C-terminus in the knock-in cells (KI), and was not observed in the parental cells that only encoded WT SREBP2. Representative of 2 validation experiments (**f**) SREBP2 processing in HeLa (WT) or HeLa SREBP2-Clover cells treated with or without StD for 40 hr. Cells were exposed to 1% oxygen for the last 16 hr as indicated. SREBP2 antibody

recognises the N-terminal region, whereas the GFP antibody detects the Clover tag. C-SRE, cleaved C-terminal region following processing in StD. (**g**) Time-course analysis of SREBP2 in HeLa SREBP2-Clover knock-in cells in 1% oxygen for up to 24 hr. Immunoblots in (**f**) and (**g**) representative of 3 independent experiments. Source data are provided as a Source Data file.

Supplementary Fig.2: Hypoxia overrides the canonical sterol-sensing response.

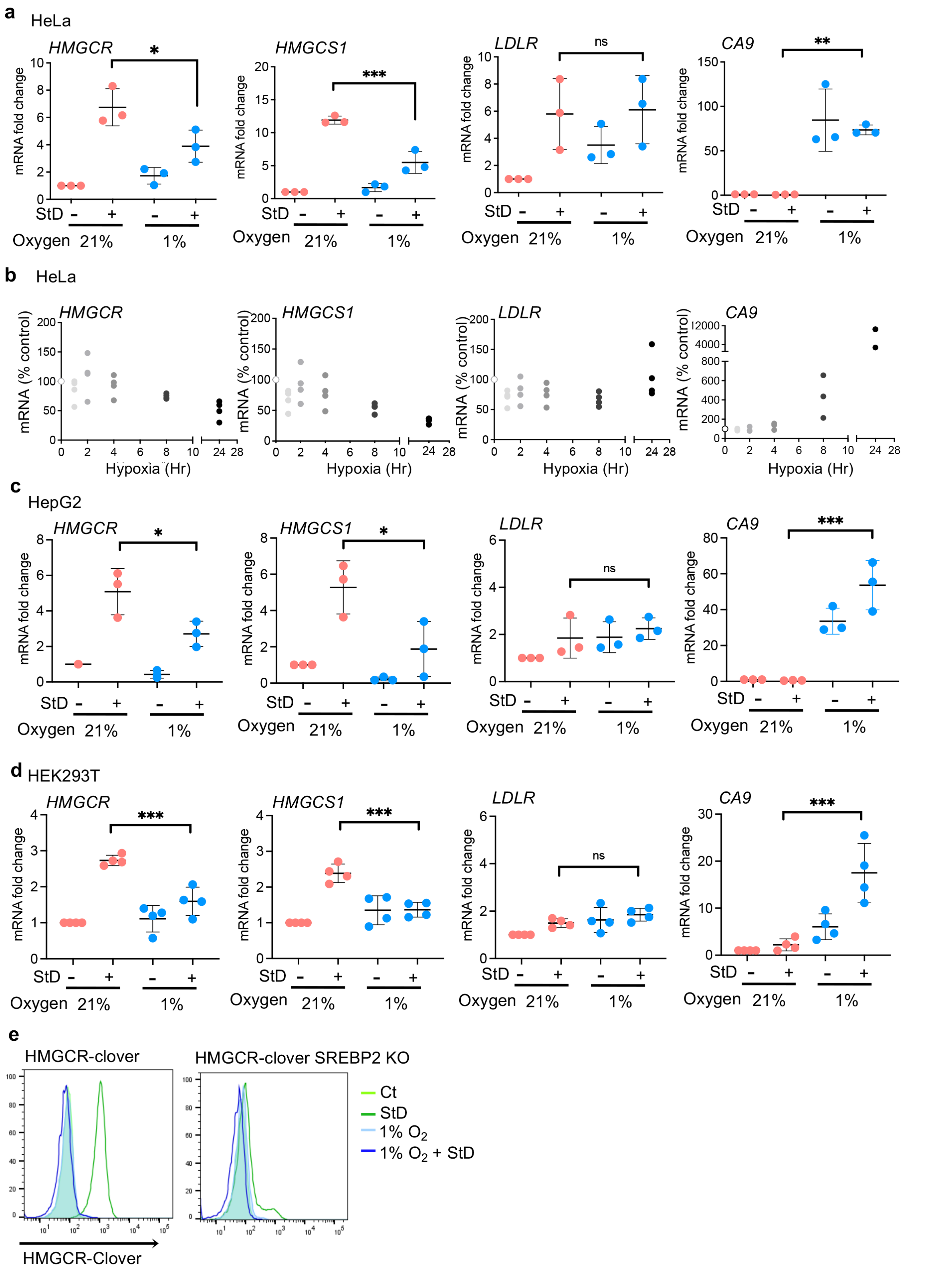

(**a**) mRNA expression of SREBP2 target genes (*HMGCR and HMGCS1*), the SREBP1 and 2 target *LDLR*, and the HIF-1 target gene *CA9* in HeLa cells treated with or without sterol depletion (StD) in 21% and 1% oxygen for 42 hr. StD 21% oxygen versus 1% oxygen *HMGCR*, *P*=0.02, *HMGCS1*, *P*<0.0001, *LDLR*, *P*=0.99, *CA9*, *P*=0.007. n=4 biological repeats except CA9 where n=3 biological repeats, mean ± SD.

*P≤0.05, **P≤0.001 Two-way ANOVA. (**b**) Time-course of mRNA expression of SREBP2 target genes (*HMGCR and HMGCS1*), the SREBP1 and 2 target *LDLR*, and the HIF-1 target gene *CA9* in HeLa cells incubated in StD conditions and 1% oxygen for up to 24 hr. n=3 biological repeats, mean ± SD. (**c, d**) mRNA expression of SREBP2 target genes (*HMGCR and HMGCS1*), the SREBP1 and 2 target *LDLR*, and the HIF-1 target gene *CA9* in HepG2 (**c**) or HEK293T cells (**d**) treated with or without StD in 21% and 1% oxygen for 42 hr. n=3 biological repeats (**c**) and 4 biological repeats (**d**), mean ± SD. *P≤0.05, ***P≤0.001 Two-way ANOVA. (**e**) Control HeLa HMGCR-clover or mixed KO populations of SREBP2 with or without StD for 42 hr. Cells were incubated in 21% or 1% oxygen for the final 18 hr. Cells were then analysed by live cell flow cytometry. Source data are provided as a Source Data file.

Supplementary Fig.3: Oxygen-mediated regulation of cholesterol synthesis is independent of HIFs

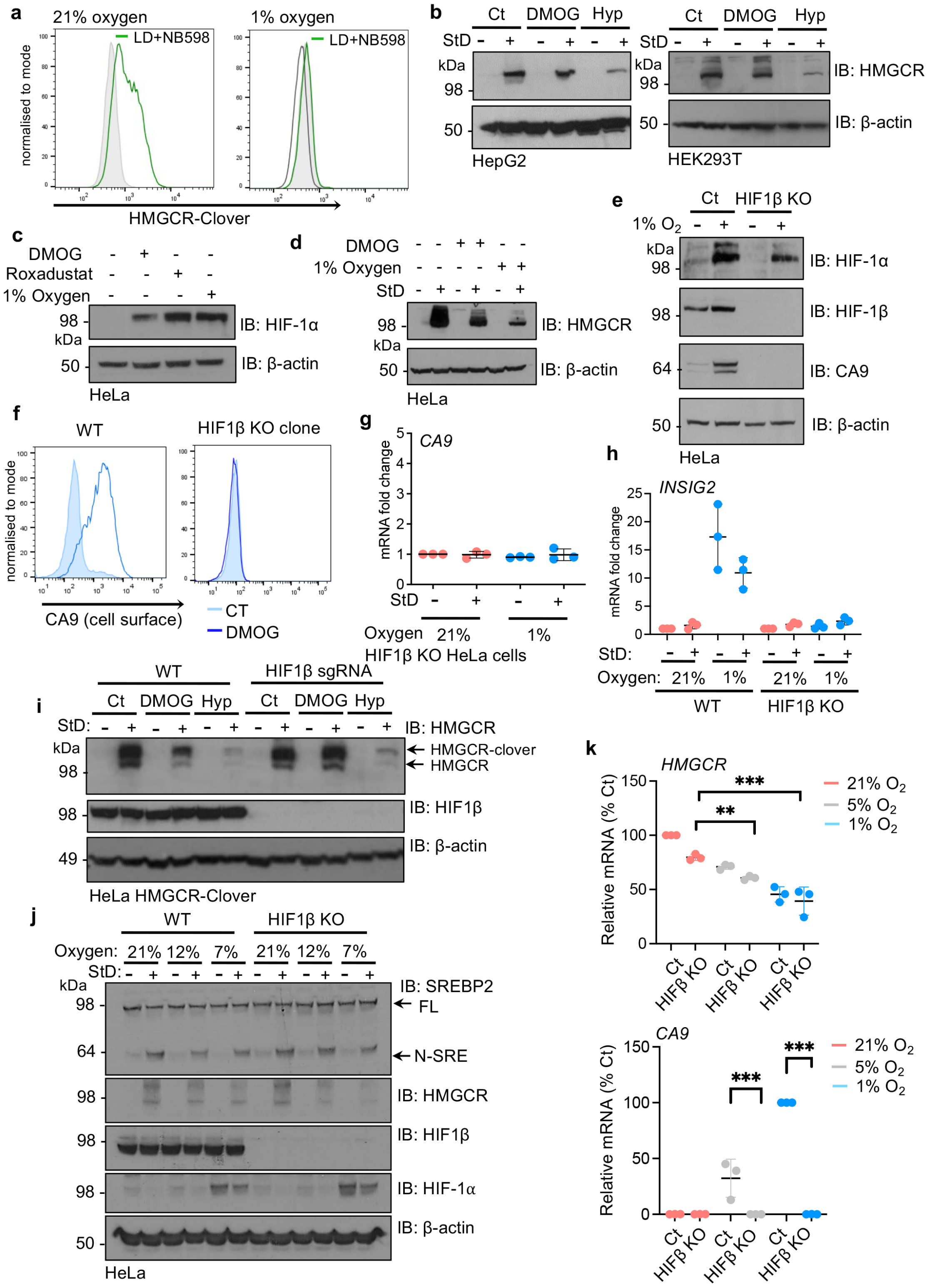

(**a**) Flow cytometry analysis of HMGCR-Clover levels following incubation in 21% or 1% oxygen, with or without 10% lipid depleted media plus the SQLE inhibitor NB-598 maleate (1 μM) for 24 hr. (**b, c**) Endogenous HMGCR levels in HepG2 cells (**c**) and HEK293T cells (**d**) following incubation in 21% (Ct), 1 mM DMOG treatment, or 1% oxygen (Hyp), with or without StD, for 24 hr. Immunoblots representative of 2 independent experiments. (**c**) HIF-1α levels in HeLa cells following treatment

with the 2-OG dependent dioxygenase inhibitor, DMOG (1 mM), the PHD inhibitor, Roxadustat (100 μM_, or incubation in 1% oxygen for 24 hr. (**d**) Endogenous HMGCR levels in HeLa cells with or without StD (40 hr) following incubation in 21%, 1 mM DMOG treatment, or 1% oxygen for the last 16 hr. (**e, f**) Confirmation of HIF1β deficient clone, with HIF-1α, HIF1β and CA9 levels after 24 hr exposure to 1% oxygen (**e**), or cell surface CA9 levels after 16 hr treatment with 1 mM DMOG (**f**). Immunoblots in (**c-e**) representative of 3 independent experiments. Flow cytometry (**f**) representative of 3 independent experiments. (**g**) mRNA levels of *CA9* (HIF-1 target gene) in HIF1β clonal KO HeLa cells following sterol depletion, with or without incubation in 1% oxygen. n=3 biological repeats, mean ± SD. (**h**) mRNA levels of INSIG2 in HIF1β clonal KO or control HeLa cells with or without sterol depletion for 24 hr, followed by incubation in 21% or 1% oxygen for 16 hr. n=3 biological repeats, mean ± SD. (**i**) HMGCR levels in control HeLa cells or following depletion of HIF1β (HIF1β sgRNA) with or without sterol depletion for 24 hr, followed by incubation in 21% or 1% oxygen, or following 1 mM DMOG treatment for 16 hr. HMGCR levels measured by immunoblot. Representative of 3 independent experiments. (**j**) SREBP2 and HMGCR levels in HeLa control or Hela HIF1β KO cells cultured in 21%, 12%, or 7% oxygen, with or without StD for 24 hr. n=3 biological repeats. (**k**) mRNA levels of HMGCR (left), and CA9 (right) in sterol depleted (24 hr) HIF1β KO or control HeLa cells following, incubated in 21%, 5% or 1% oxygen (16 hr). HMGCR mRNA HIF1β KO 21% versus 5% oxygen, $P<0.008$, HMGCR mRNA HIF1β KO 21% versus 1%, $P<0.0001$, CA9 mRNA HIF1β KO 21% versus 5% oxygen, $P<0.0001$, CA9 mRNA HIF1β KO 21% versus 1%, $P<0.0001$, n=3 biological repeats, mean ± SD. **$p\leq0.01$, ***$p\leq0.001$ Two-way ANOVA. Source data are provided as a Source Data file.

Supplementary Fig.4: Mass spectrometry analysis of [^{13}C]glucose incorporation into cholesterol isotopomers.

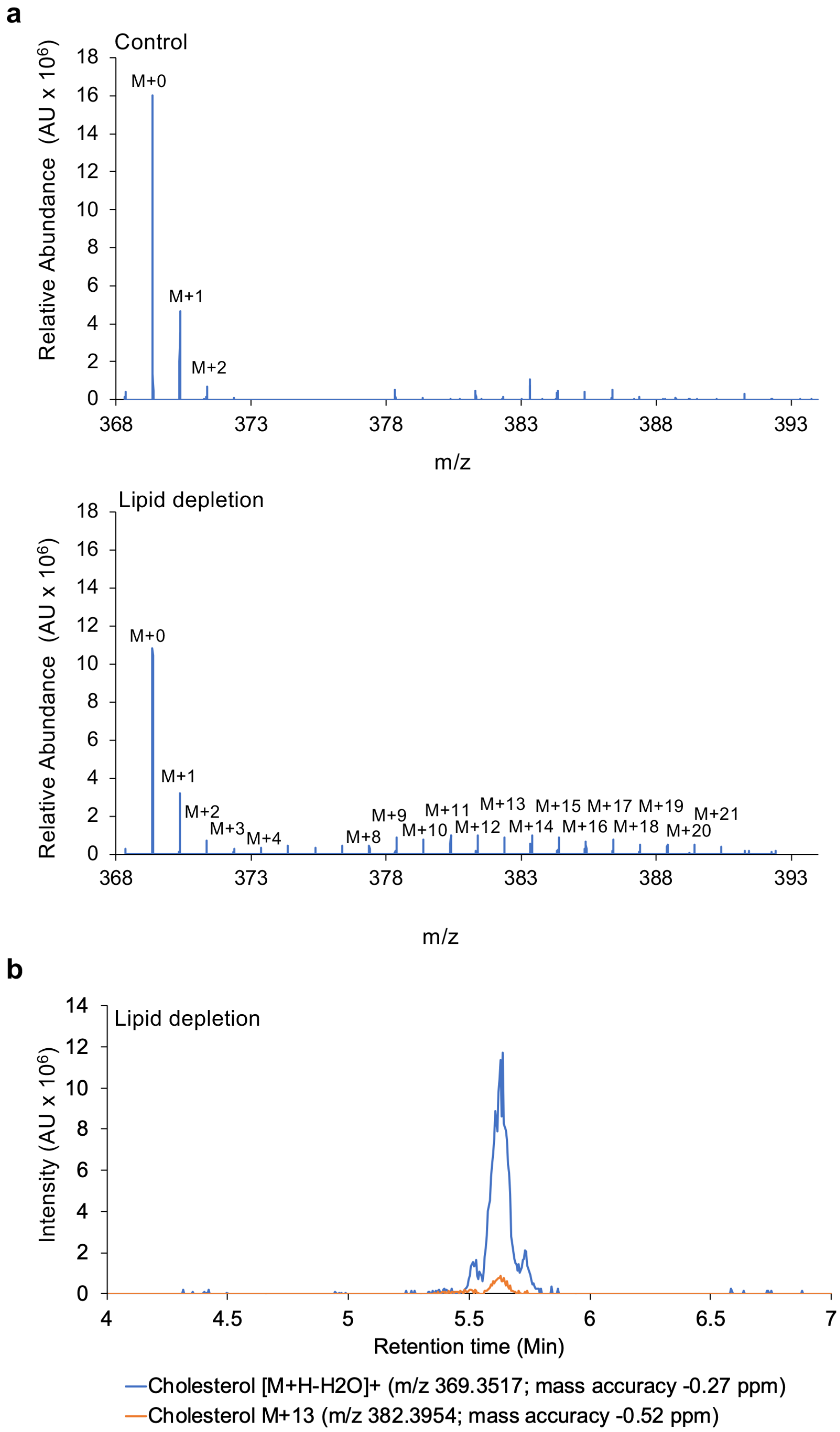

(**a, b**) Illustrative mass spectra for HeLa cells treated with (a, top panel) or without lipid deletion (a, bottom panel), and a chromatograms of unlabelled cholesterol (M+H-H_2O) and M+13 cholesterol from the lipid depleted sample (**b**). The raw data is included in Supplementary Data 4. Source data are provided as a Source Data file.

Supplementary Fig.5: The E3 ligases RNF145, GP78, Hrd1, and E2 enzyme UBE2G2 degrade HMGCR in hypoxia.

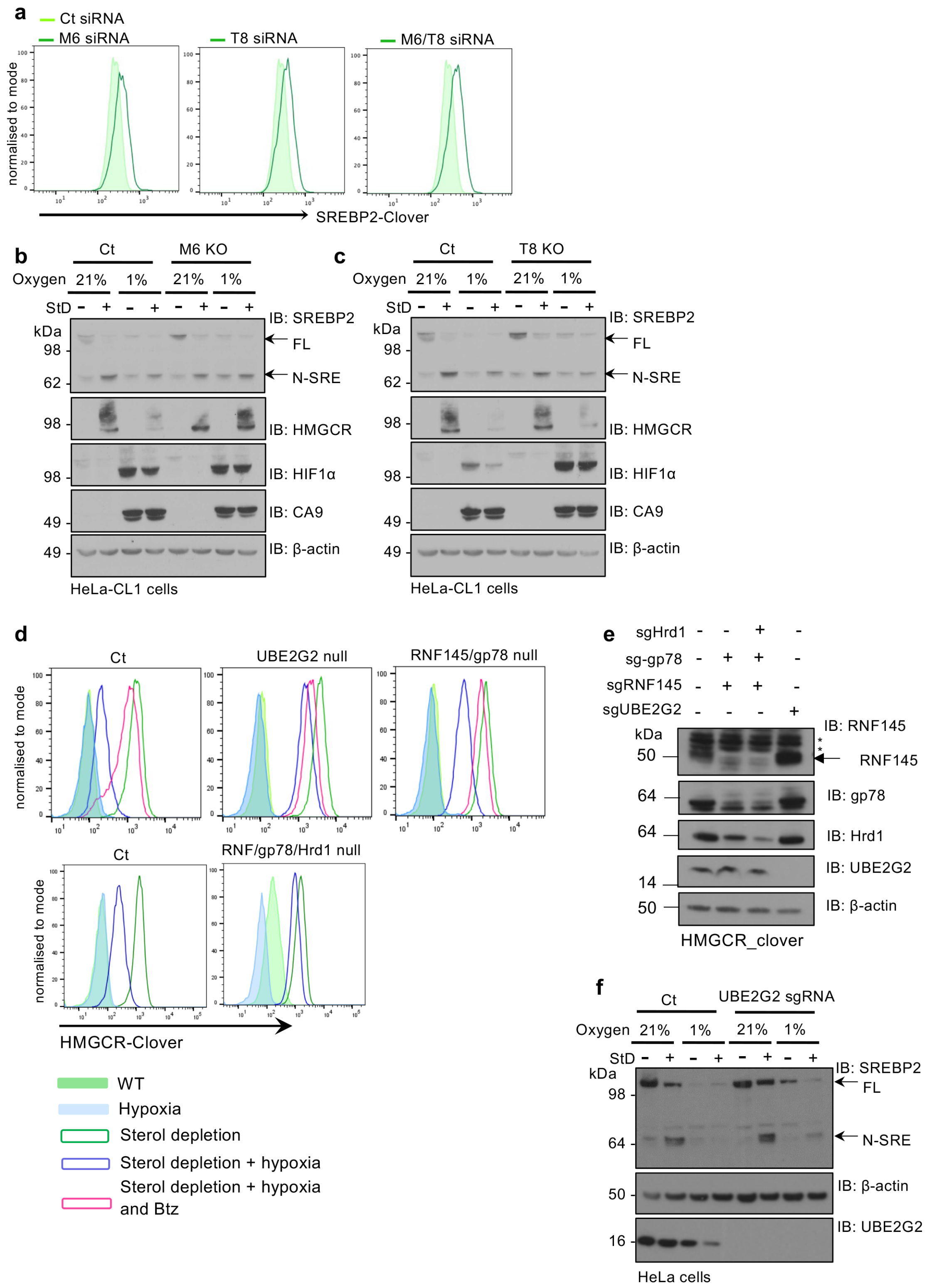

(**a**) SREP2-Clover knock-in levels, measured by flow cytometry, in HeLa cells following siRNA-mediated depletion of MARCHF6 (M6), TRC8 (T8), or combined M6/T8 depletion. The data depicted in the three panels originated from the same experiment and as such the control plot is the same in

all panels. (**b, c**) HeLa mCherry-CL1 control (Ct), MARCHF6 (**b**) or TRC8 (**c**) HeLa mCherry-CL1 clonal null cells were treated with StD for 42 hr, with or without 1% oxygen for the final 18 hr. SREBP2 processing and HMGCR levels were analysed by immunoblot. Representative of 3 independent experiments. (**d, e**) HeLa HMGCR-Clover control, UBE2G2 deficient, RNF145/gp78 deficient, or RNF145/gp78/HRD1 deficient cells were generated by sgRNA. Cells were placed under StD conditions for 42 hr, with or without hypoxia 1% oxygen for the final 18 hr. StD cells were also treated with 20nM bortezomib (Btz) for the final 18 hr. HMGCR levels were analysed by flow cytometry (**d**) or immunoblot (**e**). (**f**) SREBP2 levels in control or UBE2G2 deficient (sgRNA) HeLa cells followed StD with or without incubation in 1% oxygen as described. (**d-f**) Representative of three independent experiments. Source data are provided as a Source Data file.

Supplementary Fig.6: Transcriptional regulation of *MARCHF6* and *TRC8*

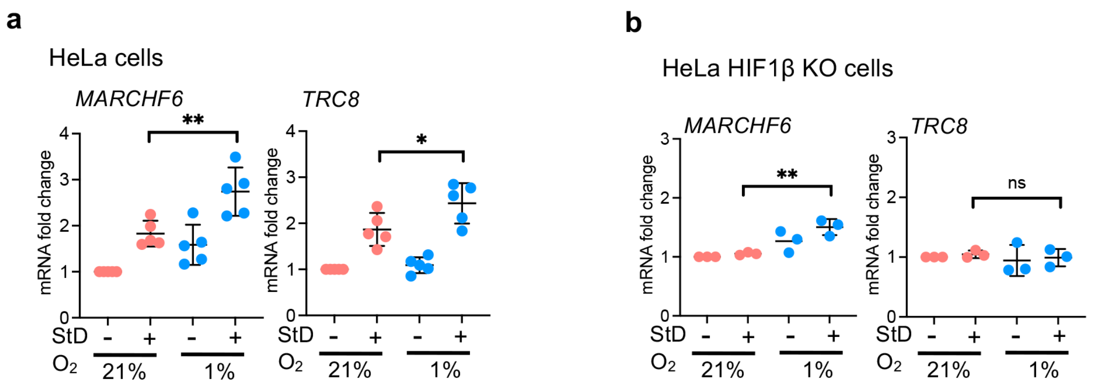

(**a, b**) HeLa control (**a**) or HIF1β null clonal cells (**b**) were sterol depleted for 42 hr, with or without 1% oxygen for the final 18 hr and *HMGCR, MARCHF6,* and *HMGCR* mRNA levels measured by qPCR. (**a**) *MARCHF6* mRNA cells StD 21% versus 1% oxygen, *P*=0.006, *TRC8* mRNA cells StD 21% versus 1% oxygen, *P*=0.04*,* (**b**) *MARCHF6* mRNA HIF1β KO cells StD 21% versus 1% oxygen, *P*=0.006, *TRC8* mRNA HIF1β KO cells StD 21% versus 1% oxygen, *P*=0.006. n=5 biological repeats (**a**) and 3 biological repeats (**b**), mean ± SD. *P≤0.05, **P≤0.01 Two-way ANOVA. Source data are provided as a Source Data file.

Supplementary Fig.7: Hypoxia promotes cholesterol auxotrophy in tumour cells by supressing cholesterol synthesis.

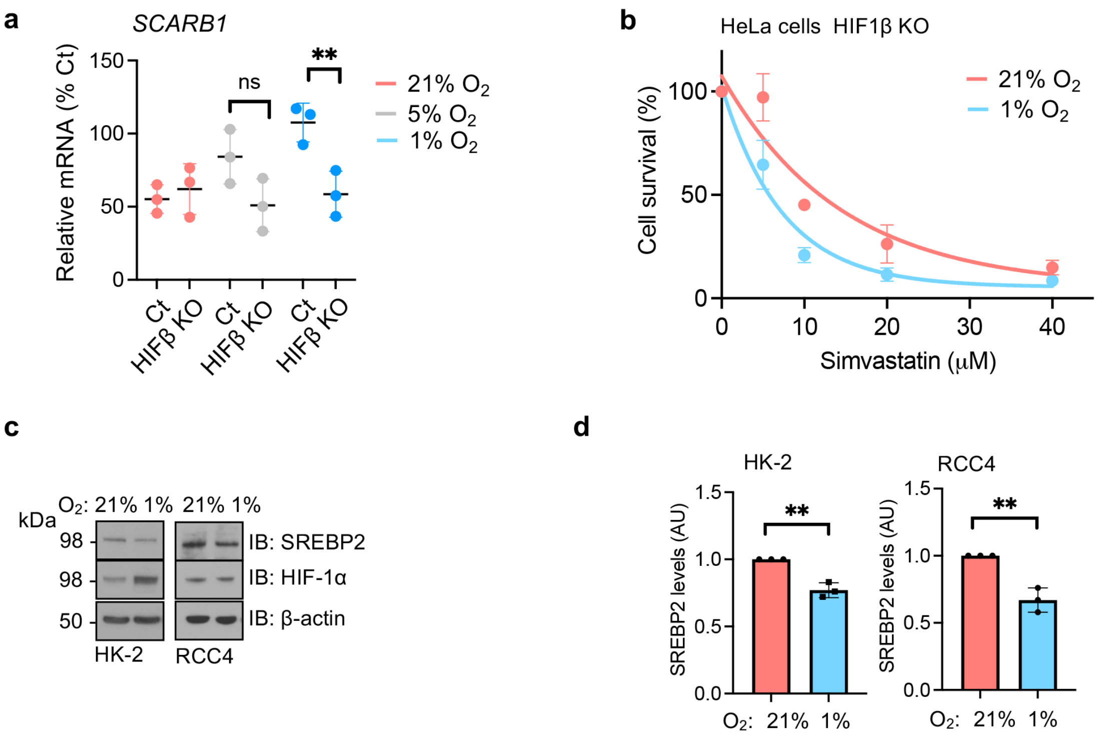

(**a**) mRNA levels of *SCARB1* in HIF1β KO or control HeLa cells with or without StD for 24 hr, followed by incubation in 21% or 1% oxygen for 16 hr. *SCARB1* mRNA cells Ct versus HIF1β KO in 5% oxygen, *P*=0.07, *SCARB1* mRNA cells Ct versus HIF1β KO in 1% oxygen, *P*=0.008. n=3 biological repeats, mean ± SD. **p≤0.01, Two-way ANOVA. (**b**) HeLa HIF1β null cells were incubated in 21% or 1% oxygen for 48 hr with simvastatin as indicated. Cell viability was measured by Cytotox staining at 48 hr. *n=3 biological repeats, mean ± SD*. (**c, d**) SREBP2 levels in HK2 or RCC4 cells incubated in 21% or 1% oxygen for 24 hr. SREBP2 levels were measured by immunoblot (**c**) and quantified using ImageJ (**d**). HK-2 cell SREBP2 protein levels in 21% versus 1% oxygen, *P*=0.002, RCC4 cell SREBP2 protein levels in 21% versus 1% oxygen, *P*=0.003. n=3 biological repeats, mean ± SD. **p≤0.01, unpaired two sample student T-Test. AU = Arbitrary Units. Source data are provided as a Source Data file.

Supplementary Table 1: Reagents

Reagents	Source	Identifier	
Antibodies			Dilution use
β-actin	Sigma Aldrich	A2228	1:20,000
HA	Covance	16B12	1:1000
HA	Roche	11867423001	1:1000
UBE2G2	Santa Cruz Biotechnology	sc-100613	1:1000
HIF1α	BD Transduction Laboratories	619959	1:1000
HIF2α	Cell Signaling	7096S	1:1000
HIF1β	Cell Signaling	5537S	1:1000
CA9 (Carbonic Anhydrase 9) M75	Absolute Antibodies	AB00414-1	1:2000
HMG CoA Reductase	Santa Cruz Biotechnology	sc-271595	1:1000
Hrd1	Abgent	AP2184a	1:1000
RNF145	ProteinTech	24524-1-AP	1:1000
gp78	ProteinTech	16675-1-AP	1:1000
Ubiquitin	Cell Signaling	3936	1:2000
SREBP2	R&D Systems	AF7119	1:1000
Anti-goat HRP	Jackson ImmunoResearch	115-35-146	1:20,000
Anti-mouse HRP	Jackson ImmunoResearch	115-035-045	1:20,000
Anti-rabbit HRP	Jackson ImmunoResearch	111-035-045	1:20,000
Anti-rat HRP	Jackson ImmunoResearch	112-35-003	1:20,000
Alexa Fluor Anti-mouse 647	Invitrogen	A11036	1:1000
Alexa Fluor Anti-mouse 488	Invitrogen	A31553	1:1000
NADK	Cell Signaling	55948	1:1000
Chemicals, peptides, and recombinant proteins			
FuGENE	Promega	E5911	
Trans-IT 293	Mirus	MIR-2705	
Trans-IT HeLa Monster	Mirus	MIR-2900	
Lipofectamine RNAiMAX	Invitrogen	13778150	
MG132	Sigma Aldrich	M8699	
Bortezomib (Velcade)	Gift from Alfred Goldberg	N/A	
Lipoprotein-deficient serum (LPDS)	Biosera	FB-1001L/10	
Digitonin	Calbiochem	300410	

Mevastatin	Sigma Aldrich	M2537	
Simvastatin	Sigma Aldrich	S6196	
DMOG	Sigma Aldrich	D3695	
Roxadustat (FG-4592)	Selleckchem	S1007	
Blasticidin	Cambridge Bioscience	14499	
Hygromycin	Cambridge Bioscience	2589-1000	
Puromycin	Cambridge Bioscience	P097	
Cycloheximide	Sigma Aldrich	01810	
Hoechst	BioTechne	5117/50	
Protoscript II reverse transcriptase	New England Biolabs	M0368	
Power SYBR Green Master Mix	Thermo Fisher Scientific	4368577	
cOmplete Protease Inhibitor Cocktail	Roche	11697498001	
Denerase	c-Lecta	20804	
Benzonase	Sigma Aldrich	E1014	
NuPAGE 4 to 12%, Bis-Tris gel	Thermo Fisher	#NP0322BOX	
Methanol	Sigma Aldrich	#34860	
[C^{13}] glucose	Sigma Aldrich	389374	
DMEM	Sigma Aldrich	D6429	
RPMI-1640	Sigma Aldrich	R8758	
MES Running Buffer	Thermo Fischer	NP0002	
ECL Supersignal	Thermo Fischer	A38556	
Supersignal West Pico PLUS	Thermo Fischer	34577	
PureLink RNA mini kit	Thermo Fisher Scientific	#12183025	
Gentra Puregene Core kit	Qiagen	#158722	
NADP+/NAPDH Assay Kit	Abcam	Ab65349	
NB-598 Maleate	Apexbio	A3647-APE	

Supplementary Table 2: Plasmids

Plasmids		
Bassik lab Human CRISPR-Cas9 deletion library	Morgens et al[1]	Addgene #101926-34
Toronto KnockOut (TKO) CRISPR Library- Version 3	Mair et al[2]	Addgene #90294
pKLV-U6gRNA-EF(BbsI) -PGKpuro2ABFP	Ochiai et al[3]	Addgene #62348
LentiCRISPRv2	Sanjana et al[4]	Addgene #52961
Lenti-Cas9-T2A-Puro	Hart et al[5]	Addgene #73310
LentiCas9-Blast	Sanjana et al[4]	Addgene #52962
pHRSIN-FLAG-NLS-Cas9-NLS-pGK-Hygro	Ortmann et al[6]	N/A
pHRSIN-pSFFV-HA-pPGK-Puro	Stefanovic-Barrett et al[7]	N/A
pMD.G (Lentiviral VSVG)	Burr et al[8]	N/A
pMD.GagPol (Lentiviral Gag/Pol)	Burr et al[8]	N/A
SREBF2 image clone 6169568	Source Bioscience	IRATp970B0781D
HA-SREBP2	This paper	N/A

Supplementary Table 3: sgRNA sequences

Gene	Sequence (5' → 3')
HIF1β	CAGTCCTCCGTCTCCTCACC
HRD1	GGTGTTCTTTGGGCAACTGA
MARCH6	TATCATCCTTGTGTATGTAC
TRC8	GCACGATGCAGAACCGGCTT
UBE2G2	CATGGGCTACGAGAGCAGCG
RNF145	TGTTAAATGTGGCCCTG
gp78	GTTAGCTGGTCCGGCTCGCC

Supplementary Table 4: Primers for generation of SREBP2 plasmids and knock-in

Primer name	Sequence (5' → 3')
HA_SREBP2_FL_fwd	GCTTATCCTTACGACGTGCCTGACTACGCCGGATCC GACGACAGCGGCGAGCTGGGTGG
FL_SREBP2 _notag_rev	GCCTGCAGGTCGACTCTAGAGTCGC TCAGGAGGCGGCAATGGCAGTG
SREBP2 5' arm for	CAGCGGCCGCGGTGCCAGGGCGTGCC CTTGGGCTCCCCGGGCGCGACTAG
SREBP2 5' arm rev	GCTCACCATGGATCCACCAGATCCGCCACCA GATCCGCCACCAGATCCGCCCGATCGG
SREBP2 3' arm for	CCAGCTGGGGCTCGAGATAA CTTCGTATAGCATACATTATACG
SREBP2 3' arm rev	GAGATCCACTAGAGTGTGGCGGCC GCATTCTTATAATCAGCATCATGATGTG
SREBP2 3' arm rev	GAGATCCACTAGAGTGTGGCGGCC GCATTCTTATAATCAGCATCATGATGTG
SREBP2_0_0 sgRNA (knock-in)	GGCTGAGCCTGGTGGTCAGG
SREBP2_1_0 sgRNA (knock-in)	GTGGGCTGAGCCTGGTGGTC
SREBP2_3_0 sgRNA (knock-in)	GTGGAGGGGTGGGCTGAGCC
SREBP2_4_0 sgRNA (knock-in)	GAAATCGAGAGAGAGGTGGA
SPfor	CACCTCTCGCCTCTCTGAGAATG
SPrev	GAGTGGGAAGGAACAGGACAATTA

Supplementary Table 5: Primers used in the mutagenesis screens

**HMGCR_Clover Bassik Library screen** Primer name	Sequence (5' → 3')
Outer_Fwd	AGGCTTGGATTTCTATAACTTCGTATAGCATACATTATAC
Outer_Rev	ACATGCATGGCGGTAATACGGTTATC
P5_inner_Fwd	AATGATACGGCGACCACCGAGATCTACACTCTCTTGTGGAAAGGACGAAACACCG
P5_index_inner_Rev	CAAGCAGAAGACGGCATACGAGAT*NNNNNNN*GTGACTGGAGTTCAGAC GTGTGCTCTTCCGATCCGACTCGGTGCCACTTTTTC
Illumina sequencing primer	AGACTATAAGTATCCCTTGGAGAACCACCTTGTTGG
**SREBP2_Clover TKOv3** Primer name	Sequence (5' → 3')
Outer_Fwd	GAGGGCCTATTTCCCATGATTC
Outer_Rev	CAAACCCAGGGCTGCCTTGGAA

Inner_Fwd	AATGATACGGCGACCACCGAGATCTACACTCTCTTGTGGAAAGGACGAGGTACCG
PCR2_inner_Rev	CAAGCAGAAGACGGCATACGAGAT*NNNNNNN*GTGACTGGAGTTCAGACGTGTGCTC TTCCGATCTATTTTAACTTGCTATTTCTAGCTCTAAAAC
Illumina sequencing primer	ACACTCTCTTGTGGAAAGGACGAGGTACCG

Supplementary Table 6: Quantitative PCR primers

Primer name	Sequence (5’ → 3’)
β-actin fw	CTGGGAGTGGGTGGAGGC
β-actin rv	TCAACTGGTCTCAAGTCAGTG
CA9 fw	GCCGCCTTTCTGGAGGA
CA9 rv	TCTTCCAAGCGAGACAGCAA
HMGCR fw	CGTGGAATGGCAATTTTAGGTCC
HMGCR rv	ATTTCAAGCTGACGTACCCCT
HMGCS1 fw	GATGTGGGAATTGTTGCCCTT
HMGCS1 rv	ATTGTCTCTGTTCCAACTTCCAG
INSIG2 fw	TAATGCGGTGTGTAGCAGTCT
INSIG2 rv	GTCCAATGGATAGTGCAGCCA
LDLR fw	ACCAACGAATGCTTGGACAAC
LDLR rv	ACAGGCACTCGTAGCCGAT
SREBP2 fw	AACGGTCATTCACCCAGGTC
SREBP2 rv	GGCTGAAGAATAGGAGTTGCC
SCARB1 fw	CCTATCCCCTTCTATCTCTCCG
SCARB1 rv	GGATGTTGGGCATGACGATGT
MARCHF6 fw	CACCTGAGAAACCGCTTTA
MARCHF6 rv	CCGTGAAGGCATATCTGGAGA
TRC8 fw	ATCGACGCCATCTTCAACTCC
TRC8 rv	AAGCTGACGTGTTGTAGGCAC

Supplementary References.

1 Morgens, D. W. *et al.* Genome-scale measurement of off-target activity using Cas9 toxicity in high-throughput screens. *Nature communications* **8**, 15178, doi:10.1038/ncomms15178 (2017).

2 Mair, B. *et al.* Essential Gene Profiles for Human Pluripotent Stem Cells Identify Uncharacterized Genes and Substrate Dependencies. *Cell reports* **27**, 599-615.e512, doi:10.1016/j.celrep.2019.02.041 (2019).

3 Ochiai, H., Sugawara, T. & Yamamoto, T. Simultaneous live imaging of the transcription and nuclear position of specific genes. *Nucleic Acids Res* **43**, e127, doi:10.1093/nar/gkv624 (2015).

4 Sanjana, N. E., Shalem, O. & Zhang, F. Improved vectors and genome-wide libraries for CRISPR screening. *Nature methods* **11**, 783-784, doi:10.1038/nmeth.3047 (2014).

5 Hart, T. *et al.* High-Resolution CRISPR Screens Reveal Fitness Genes and Genotype-Specific Cancer Liabilities. *Cell* **163**, 1515-1526, doi:10.1016/j.cell.2015.11.015 (2015).

6 Ortmann, B. M. *et al.* The HIF complex recruits the histone methyltransferase SET1B to activate specific hypoxia-inducible genes. *Nature genetics* **53**, 1022-1035, doi:10.1038/s41588-021-00887-y (2021).

7 Stefanovic-Barrett, S. *et al.* MARCH6 and TRC8 facilitate the quality control of cytosolic and tail-anchored proteins. *EMBO Rep* **19**, doi:10.15252/embr.201745603 (2018).

8 Burr, S. P. *et al.* Mitochondrial Protein Lipoylation and the 2-Oxoglutarate Dehydrogenase Complex Controls HIF1alpha Stability in Aerobic Conditions. *Cell metabolism* **24**, 740-752, doi:10.1016/j.cmet.2016.09.015 (2016).
